# Sexual dysfunctions and short-term glucose variability in young men with type 1 diabetes

**DOI:** 10.1007/s42000-021-00295-1

**Published:** 2021-04-30

**Authors:** Paola Caruso, Paolo Cirillo, Carla Carbone, Annalisa Sarnataro, Maria Ida Maiorino, Giuseppe Bellastella, Katherine Esposito

**Affiliations:** 1 Department of Advanced Medical and Surgical Sciences, University of Campania “Luigi Vanvitelli”, Piazza L. Miraglia 2, 80138 Naples, Italy; 2 Division of Endocrinology and Metabolic Diseases, University of Campania “Luigi Vanvitelli”, Naples, Italy; 3 Unit of Diabetes, University of Campania “Luigi Vanvitelli”, Naples, Italy

**Keywords:** Type 1 diabetes, Glucose variability, Erectile dysfunction, Premature ejaculation, Sexual dysfunction

## Abstract

**Purpose:**

Erectile dysfunction (ED) and premature ejaculation (PE) are common sexual disorders in people with diabetes. Glucose variability (GV) has been recognized as a predictor of microvascular complications. The aim of this study was to investigate the relationship between glucose variability and sexual dysfunctions in young men with type 1 diabetes.

**Methods:**

One hundred and twelve patients with type 1 diabetes, aged 18–30 years, were enrolled. Patients were divided into two groups according to glucose variability [group 1 (high GV with coefficient of variation ≥ 36%)] and group 2 (low GV with coefficient of variation < 36%)). The presence of sexual dysfunctions was investigated with validated questionnaires.

**Results:**

ED and PE prevalence rates in group 1 were 26% and 13%, respectively. Similarly, in group 2, the prevalence of ED was 24%, and the prevalence of PE was 13%. In both groups, no significant associations between sexual dysfunctions and parameters of glucose variability were found. Multiple regression analysis identified age and depression as independent predictors of ED and PE.

**Conclusion:**

Young male patients affected by type 1 diabetes with high or low glucose variability show a similar prevalence of sexual dysfunctions. ED is the most common sexual dysfunction in diabetic men. Age and depression were the only independent predictive factors for sexual dysfunctions in this population.

## Introduction

Diabetes is one of the most common chronic diseases in nearly all countries worldwide and a well-known risk factor for sexual dysfunctions in men [1]. Erectile dysfunction (ED) is defined as the persistent or recurrent inability to achieve and/or maintain penile erection sufficient for satisfactory sexual performance [2]. ED is a common sexual arousal disorder in men with diabetes [3], a threefold increased risk having been documented in the Massachusetts Male Aging Study [4] as compared to nondiabetic men. ED generally occurs 10–15 years sooner in men with diabetes [4] than in those who do not suffer from diabetes and is more severe [5] and less responsive to oral drugs [6, 7], leading to a reduction in quality of life [5, 8]. Whether hyperglycemia is a risk factor for the development of ED in diabetic men is still not clear. Some observational studies have shown a relationship between poor glycemic control, expressed by elevated levels of glycated hemoglobin (HbA_1c_) and ED [5, 9, 10], whereas other studies did not report any association [11].

While ED is a well-known diabetes-related sexual dysfunction, ejaculatory and sexual desire issues may also occur in men. Premature ejaculation (PE) is the most frequent male sexual dysfunction, although the true prevalence of this sexual disorder is unclear. The second International Society of Sexual Medicine Ad Hoc Committee for the Definition of Premature Ejaculation [12] defined PE (lifelong and acquired) as a male sexual dysfunction characterized by an ejaculation that occurs sooner than desired after vaginal penetration on all or most occasions, or inability to delay ejaculation during sexual intercourse with generation of individual distress, bother, and frustration that negatively impact on the couple’s relationship. In several observational studies, the prevalence of PE has been reported to be higher in diabetic men as compared to healthy controls [13, 14]. Moreover, diabetic people were found to have a higher incidence of PE with increased severity of ED compared to the general population [13, 15].

The correlation between glycemic control and microvascular complications is well known in both type 1 and type 2 diabetes [12, 16–20]. Moreover, glycemic variability (GV), defined as the frequency and amplitude of glycemic excursions around the mean of glucose values, expressed using standard deviation or other indices, has emerged as an independent predictor of these complications [21–23]. Indeed, GV has been associated with poor glycemic control, poor quality of life, and increased risk of diabetes-related complications [24].

To the best of our knowledge, there are no studies evaluating the role of GV in sexual dysfunctions in men with type 1 diabetes. Therefore, the aim of the present study was to assess the relationship between GV and sexual dysfunctions in young men with type 1 diabetes.

## Materials and methods

### Participants

This is a single-center, cross-sectional study aimed at evaluating the influence of GV on sexual dysfunctions in young men with type 1 diabetes admitted to the Unit of Diabetes at the Teaching Hospital of the University of Campania “Luigi Vanvitelli,” Naples, Italy. From January to June 2020, men were included in the study if they (1) were aged ≥ 18 and ≤ 30 years, (2) had stable couple relationship or sexual activity (masturbation) in the previous month, and (3) did not use phosphodiesterase type 5 inhibitors (PDE5-i). Exclusion criteria were considered the presence of any chronic diseases not including diabetes complications (neoplasms, severe neurodegenerative diseases, major depression or other psychiatric disorders, hypogonadism, penis disorders, drug or alcohol abuse), the use of drugs associated with adverse effects on erectile function, a history of urological surgery, lower urinary tract symptoms, and pelvic trauma in the last 6 months.

### Assessment of sexual function

All participants in the study were asked to complete the Italian version of three different validated multiple-choice self-reported questionnaires assessing both erectile and ejaculatory functions and the presence of depressive symptoms. Participants in the study received a short explanation in order to answer each questionnaire in the context of the visit to our diabetes unit.

Erectile function was investigated by completing the abbreviated form of the International Index of Erectile Function (IIEF-5) [25], which comprises items 2, 4, 5, 7, and 15 of the full scale IIEF-15 and assures simplicity and immediacy in its compilation. According to the recommended scoring system, a total score of 21 or less indicates the presence of ED. ED was classified as mild with a score ranging from 21 to 17, mild to moderate with a score ranging from 16 to 12, moderate with a score ranging from 11 to 8, and severe with a score lower or equal to 7.

In accordance with the European Association of Urology guidelines [26], we used the five-item premature ejaculation diagnostic tool (PEDT) [27] to assess PE and investigate control of ejaculatory function, frequency, minimal stimulation, and both distress and interpersonal difficulty. A score of 8 or lower excluded a diagnosis of PE.

### Evaluation of depressive symptoms

The 13-item short form of the Beck Depression Inventory (BDI) [28] was used to assess the presence of depressive symptoms. This self-report questionnaire covers affective, psychological, and somatic symptoms associated with depression. Each item is scored ranging from 0 to 3. Four ranges were identified: normal (< 10), mildly depressed (10–19), moderately depressed (20–29), and severely depressed (> 30).

### Assessment of glucose variability

All patients underwent for 14 days a blinded continuous glucose monitoring (DexCom G5 CGM system—Dexcom Inc., San Diego, USA), composed of a 7-day transcutaneous sensor, a transmitter, and a receiver. The sensor was implanted in the anterior abdominal wall and changed after the first 7 days by the patients. Moreover, the men were instructed to perform the sensor calibration procedure, according to the manufacturer’s instructions, within 2 h from placing the sensor, and then every 12 h. Glucose data were downloaded with Dexcom CLARITY and analyzed by displaying the ambulatory glucose profile (AGP). We assessed GV by collecting the coefficient of variation (CV) from AGP. CV was estimated as the ratio of the standard deviation of glucose values and mean glucose multiplied per 100; values equal to or above 36% indicated high GV [29]. We also calculated the time in range (TIR) as the percentage of time spent in the glucose range between 70 and 180 mg/dL. Moreover, mean daily glucose values and standard deviation (Stdev) were also collected.

### Anthropometric measures and laboratory analyses

The height and weight of each participant were measured using a Seca 200 scale (Seca, Hamburg, Germany) with an annexed stadiometer. Body mass index (BMI) was calculated as weight (in kilograms) divided by height (expressed in meters squared). Waist circumference was also measured. At the end of clinical examination, arterial blood pressure was measured three times while subjects were sitting after 15 min resting. Patients whose average blood pressure levels were equal to or higher than 140/90 mmHg or who used antihypertensive medication were classified as affected by hypertension.

Assays for fasting glucose, HbA_1c_, total cholesterol, low-density (LDL) and high-density (HDL) lipoprotein cholesterol, triglyceride levels, and testosterone were performed in the hospital’s chemistry laboratory.

### Statistical analysis

Sample size calculation was made on the basis of a previous study investigating the relationship between sexual function, measured as Female Sexual Function Index (FSFI) total score, and GV in a population of young women with type 1 diabetes [30]. With an expected Pearson correlation coefficient between the IIEF-5 score and the CV of 0.3, a sample size of 102 patients was required assuming a power of 0.80%, a 20% rate of non-responders, and a level of significance of 0.05. Data in tables and figures concerning normally distributed variables are presented as mean ± SD, while non-normally distributed continuous variables are presented as median (interquartile range). Differences between groups were evaluated by the two-sided Student’s *t*-test or Wilcoxon-Mann–Whitney test. The χ^2^-test was used to compare dichotomous variables. Statistical associations between normally distributed variables were assessed using Pearson’s correlation test. Multivariable regression analysis tested the contribution of independent variables (age, duration of diabetes, weight, BMI, waist circumference and BDI) to the dependent variable (IIEF-5 score and PEDT score). Two-sided *P* values < 0.05 were considered statistically significant. All statistical analyses were performed using SPSS software.

## Results

**Fig. 1:**
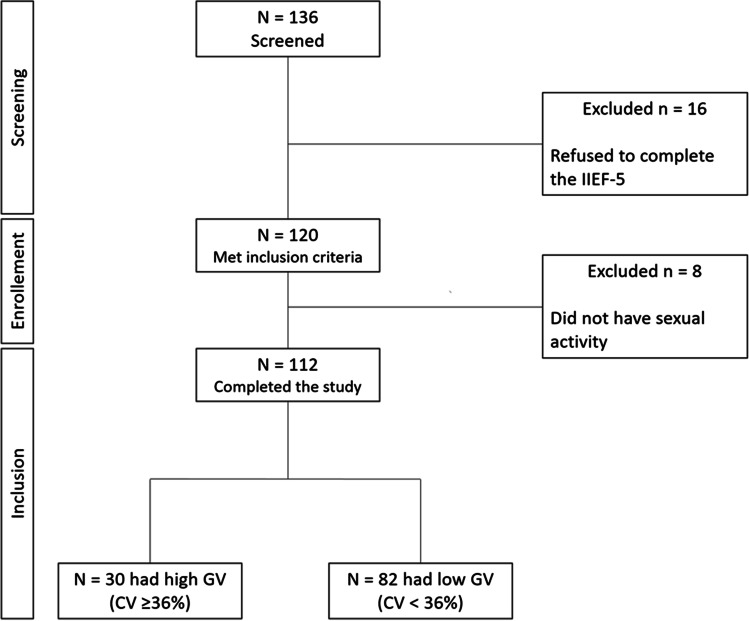
Process of patients’ selection. CV, coefficient of variation; IIEF-5, international index of erectile function

One hundred and thirty-six patients were considered eligible to be included in the study; 16 men refused to complete the IIEF-5 questionnaire and eight of the remaining 120 men were excluded because they were not sexually active. Therefore, the study population consisted of 112 type 1 diabetic men (Fig. 1). The clinical and metabolic characteristics of participants in the study are described in Table 1. The mean age was 25.8 years, and the mean duration of diabetes was 14 years; 47% of the overall population had a BMI ≥ 25 kg/m^2^, and 76% had HbA_1c_ > 7%. Sixty-eight men were treated with multiple daily injections of insulin (MDI), while the remaining 44 patients were on continuous subcutaneous insulin infusion (CSII). Compared with men on CSII, those on MDI received a higher daily insulin dose. Nine out 112 men (9%) had microvascular complications, of which the most frequent was diabetic retinopathy, which was found in six men. The overall prevalence rates of ED and PE were 25% and 13%, respectively. Among men with microvascular complications, six (67%) had ED and three (33%) had PE.

**Table 1:** Baseline characteristics of diabetic patients

Parameters	Patients with type 1 diabetes (*n* = 112)
Age, years	25.8 ± 7.5
Diabetes duration, years	14 ± 6.7
Weight, kg	76 ± 11.8
BMI, kg/m^2^	25 ± 3.2
Waist circumference, cm	83.8 ± 8.9
BMI ≥ 25 kg/m^2^, *n* (%)	53 (47)
FG, mg/dL	168 ± 69.5
HbA_1c_, %	7.8 ± 1.2
HbA_1c_ > 7, *n* (%)	85 (76)
HR, bpm	80.1 ± 6.1
SBP	125 (120, 130)
DBP	80 (70, 80)
Total cholesterol	160.8 ± 31.9
HDL cholesterol	51.5 ± 11
LDL cholesterol	97.3 ± 27.1
Triglyceride	93 ± 61.7
Testosterone, (nmol/L)	23.6 ± 5.2
Microvascular complications, *n* (%)	9 (8)
Autoimmune diseases, *n* (%)	14 (12.5)
MDI/CSII, *n*	66/44
Insulin dose, UI per day	51.3 ± 15.1
Insulin dose, UI/kg	0.68 ± 0.18
IIEF-5	22 ± 3.5
PEDT	3.5 ± 3.5
BDI	2.2 ± 2.1
CV, %	32.5 ± 11.4
CV ≥ 36, *n* (%)	23 (26.1)
TIR (%)	68.7
ED, n (%)	28 (25)
PE, n (%)	13 (11.6)
Mean daily glucose, mmol/L	10 ± 2.5
Stdev, mmol/L	3.2 ± 1.2

Data are expressed as mean ± SD, median (interquartile range), or number and percentage. Abbreviations: *BDI* beck depression inventory, *BMI* body mass index, *CSII* continuous subcutaneous insulin infusion, *CV* coefficient of variation, *DBP* diastolic blood pressure, *ED* erectile dysfunction, *FG* fasting glucose, *HDL* high density lipoprotein, *HR* heart rate, *IIEF-5* international index of erectile function-5, *LDL* low density lipoprotein, *MDI* multiple daily injection, *PE* premature ejaculation, *PEDT* premature ejaculation diagnostic tool, *SBP* systolic blood pressure, *Stdev* standard deviation of mean glucose

Twenty-six percent of diabetic men had high GV (CV ≥ 36%, group 1). There were no differences between men with high (group 1) and low GV (group 2) in all the studied clinical variables, except for CV (group 1 vs group 2, 47.6 ± 9.2 vs 27.1 ± 6.2, *P* < 0.001), TIR (60.4 ± 7.4 vs 75.6 ± 6.9, *P* < 0.001), and Stdev [4.5 (3.7, 5.4) vs 2.7 (2.2, 3.2), *P* < 0.001], which, as expected, were higher in group 1 than in group 2 (Table 2). ED and PE prevalence rates in group 1 were 26% and 13%, respectively. Similarly, in group 2 (diabetic men with low GV), the prevalence of ED was 24%, and the prevalence of PE was 13%. Among patients with microvascular complications, one man in group 1 and two men in group 2 had both ED and PE.

**Table 2:** Characteristics of diabetic patients with high (group 1, CV ≥ 36%) and low GV (group 2, CV < 36%)

Variable	Group 1 (*n* = 23)	Group 2 (*n* = 89)	*P*
Age, years	24.1 ± 6.7	26.3 ± 7.6	0.226
Diabetes duration, years	12.1 ± 5.7	14.5 ± 6.8	0.129
Weight, kg	74.5 ± 8.8	76.4 ± 12.5	0.512
BMI, kg/m^2^	24.7 ± 2.9	25.1 ± 3.4	0.623
WC, cm	83.1 ± 8.4	84.1 ± 9.1	0.685
FG, mg/dL	189 ± 72	163 ± 68	0.139
HbA1c, %	7.8 ± 1.2	7.8 ± 1.3	0.989
Testosterone, nmol/L	23.9 ± 4.3	23.2 ± 4.2	0.480
Microvascular complications, *n* (%)	2 (8.7)	7 (7.8)	0.764
CV, %	47.6 ± 9.2	27.1 ± 6.2	< 0.001
TIR, %	60.4 ± 7.4	75.6 ± 6.9	< 0.001
Mean daily glucose, mmol/L	9.5 (8.3, 10.1)	9.5 (8.3, 11.7)	0.515
Stdev	4.5 (3.7, 5.4)	2.7 (2.2, 3.2)	< 0.001
IIEF-5 score	23 (21, 24)	23 (20, 25)	0.669
Men with ED, *n* (%)	6 (26)	22 (25)	0.893
PEDT score	2 (1, 4)	3 (1, 6)	0.534
Men with PE, *n* (%)	3 (13)	10 (11)	0.901
BDI	2 (1, 3)	2 (1, 3)	0.679

Data are expressed as mean ± SD, median (interquartile range), or number and percentage. Abbreviations: *BDI* beck depression inventory, *BMI* body mass index, *CV* coefficient of variation, *ED* erectile dysfunction, *FG* fasting glucose, *IIEF-5* international index of erectile function-5, *PE* premature ejaculation, *PEDT* premature ejaculation diagnostic tool, *Stdev* standard deviation of mean glucose, *TIR* time in range, *WC* waist circumference

Correlation coefficients between metabolic and psychosocial variables in diabetic men are reported in Table 2. IIEF-5 score was negatively correlated with age (*r* =  − 0.8; *P* ≤ 0.01), duration of diabetes (*r* =  − 0.5; *P* ≤ 0.01), weight (*r* =  − 0.2; *P* = 0.02), BMI (*r* =  − 0.2; *P* = 0.02), waist circumference (*r* =  − 0.4; *P* ≤ 0.01), and BDI (*r* =  − 0.7; *P* ≤ 0.01). Likewise, we found a significant association between PEDT score and age (*r* = 0.7; *P* ≤ 0.01), duration of diabetes (*r* = 0.4; *P* ≤ 0.01), and BDI (*r* = 0.5; *P* ≤ 0.01) (Table 3). No significant associations between sexual dysfunctions and parameters of GV were observed.

**Table 3:** Correlation between IIEF-5 score, PEDT score, and metabolic and psychological domains in type 1 diabetic patients

	IIEF-5 score		PEDT score	
	Correlation coefficient (r_p_)	*P* value	Correlation coefficient (r_p_)	*P* value
Age	− 0.861	< 0.001	0.675	< 0.001
Diabetes duration	− 0.503	< 0.001	0.373	< 0.001
Weight	− 0.241	0.023	0.105	0.269
BMI	− 0.236	0.029	0.032	0.741
WC	− 0.397	< 0.001	0.129	0.217
Fasting glucose	− 0.014	0.899	0.035	0.739
HbA_1c_	− 0.028	0.786	− 0.031	0.744
BDI	− 0.721	< 0.001	0.539	< 0.001
Mean	− 0.061	0.613	− 0.024	0.824
Stdev	− 0.013	0.911	− 0.032	0.764
CV	0.043	0.717	− 0.035	0.742
TIR	0.089	0.421	− 0.074	0.459

Abbreviations: *BDI* beck depression inventory, *BMI* body mass index, *CV* coefficient of variation, *FG* fasting glucose, *LDL* low density lipoprotein, *Stdev* standard deviation, *TIR* time in range, *WC* waist circumference

In the multiple regression analysis (Table 4), in which IIEF-5 score and PEDT score were the dependent variables, only age and BDI score resulted as independent predictors of IIEF-5 score (β coefficient =  − 0.322, *P* < 0.001 and β coefficient =  − 0.569, *P* ≤ 0.001, respectively) and PEDT score (β coefficient = 0.302, *P* < 0.001 and β coefficient = 0.567, *P* ≤ 0.001, respectively).

**Table 4:** Multiple regression analysis

	IIEF-5 score		PEDT score	
	β coefficient	*P* value	Β coefficient	*P* value
Age	− 0.322	< 0.001	0.302	< 0.001
Diabetes duration	− 0.032	0.361	0.033	0.487
Weight	− 0.023	0.551	− 0.021	0.653
BMI	− 0.196	0.135	0.128	0.444
WC	− 0.049	0.241	− 0.162	0.006
BDI	− 0.569	< 0.001	0.567	< 0.001

Abbreviations: *BDI* beck depression inventory, *BMI* body mass index, *WC* waist circumference

## Discussion

To the best of our knowledge, this is the first study evaluating the prevalence of sexual dysfunctions in a population of young type 1 diabetic men with high or low GV. We found that the prevalence of sexual dysfunctions in diabetic men with high GV was comparable to that of diabetic men with low GV. Moreover, ED was observed to be the most common sexual dysfunction in this population. The young age of the study participants, the relatively large number of men included (*n* = 112), and the possibility to differentiate between men with high and low GV are distinctive characteristics of this study.

Literature data on the prevalence of sexual dysfunctions in young men affected by type 1 diabetes are scant. Moreover, the lack of any other study describing sexual function in diabetic patients according to the degree of GV does not enable comparisons to be made.

GV represents an emerging determinant of vascular complications associated with diabetes [24]. One possible reason for the absence of any association between GV and sexual dysfunction in the included population may concern the measurement of glucose fluctuation in a short period of time. Indeed, the CV for glucose has been accepted as the most appropriate index for assessing the within-day glycemic variability, with a cut-off threshold value of 36% to differentiate stable from labile glucose control [29]. At present, there is no evidence that short-term GV is an independent risk factor for cardiovascular complications in diabetes. Moreover, whether long-term GV, measured as monthly or quarterly changes in either HbA1c, fasting, or postprandial plasma glucose is associated with sexual disorders in men with type 1 diabetes, remains unknown.

Evidence linking glycemic control to sexual dysfunctions in men with type 1 diabetes is limited. Longitudinal analyses from the follow-up study of the Diabetes Control and Complications Trial (DCCT) suggest that glycemic control, together with age and BMI, is a predictor of ED [31]. In the same study, men who never reported ED or in only 1 isolated year of the follow-up had better glycemic control than men who had intermittent and persistent ED at DCCT baseline [31]. Moreover, in men with long-standing type 1 diabetes, elevated HbA1c levels were associated with sexual dysfunctions [32]. Whether the improvement of glycemic control may help prevent sexual dysfunction or restore erectile function in type 1 diabetes remains controversial [31, 33].

Diabetes, together with overweight/obesity, metabolic syndrome, and hyperlipidemia, are well-known risk factors for ED [34]. Of note, 47% of the overall diabetic population was overweight, suggesting the importance of metabolic factors in the pathogenesis of erectile complaints even in young men. On the other hand, the risk factors for PE were investigated to a lesser extent than ED. There is evidence from a cross-sectional study of young diabetic men with PE that a higher GV in the hypoglycemic domain may be significantly associated with the severity of this sexual complaint according to the calculated PEDT score [35]. However, confirming previous evidence [10], only age and depression were found to be an independent predictor of sexual function in the present study, highlighting that psychological rather than biological factors may play a major role in development of sexual dysfunctions even in young men with type 1 diabetes.

PE is known to be the most frequent sexual dysfunction in men, affecting nearly 23% of individuals worldwide and without significant variation in age among men over 24 years [14]. On the other hand, the prevalence of ED in men younger than 40 years ranges from 1 to 10% [3]. Our data show that the prevalence of ED is higher than that of PE in participants in the study, suggesting that among people with diabetes, there would be an inverse prevalence trend in sexual dysfunctions as compared to the general population. It is well known that ED may occur earlier in diabetes [4]. Moreover, both PE and ED were self-reported based on validated questionnaires, and this cannot fully substitute for physician-patient interaction and clinical examination in obtaining a diagnosis. Other potential reasons for this discrepancy are the possibility that younger men presenting short latencies may have a higher threshold for defining rapid ejaculation as a lack of ejaculatory control [14].

Age has been suggested as an important risk factor for sexual dysfunction in men. A cross-sectional analysis of 2126 adult male NHANES participants reported that in men at least 20 years old, ED affected almost one in five respondents to the survey, with prevalence rates increasing dramatically with advanced age [36]. On the other hand, younger individuals do not appear to be at higher risk for PE than older individuals up to the age of 59 [14].

Early adulthood is characterized by psychosocial challenges, which may be increased by the presence of diabetes [37]. Psychological disorders are also associated with male sexual dysfunctions. The association of depression with ED is well-established, both in the general population [38] and in diabetic patients [39]. Previous studies have shown that sexual function is more closely linked with depression than other diabetic complications in men and women [40]. Moreover, in several observational studies, men with PE were more likely to self-report both depression and anxiety [14, 41]. Interestingly, in 1206 men diagnosed with PE, depression was associated with PE duration and IIEF-5 scores lower than 22 [42].

This study has a number of limitations. First, due to its cross-sectional nature, we are not able to draw conclusions regarding cause and effect. Second, we did not distinguish between lifelong and acquired PE. Third, the presence of sexual dysfunctions was self-reported, and this could have introduced some bias. Major strengths of this study include the use of a validated tool for the evaluation of sexual dysfunction, the relatively large number of subjects investigated, and the contemporary evaluation of many aspects of life linked to sexual function (depression and general health status).

In conclusion, young male patients affected by type 1 diabetes with high or low GV show a similar prevalence of sexual dysfunctions. ED was shown to be the most common sexual dysfunction in diabetic men. Age and depression were the only independent predictive factors for sexual dysfunctions in this selected population. The role of glycemic control in the pathogenesis of sexual dysfunctions in men with type 1 diabetes remains to be clarified. Meanwhile, evaluation of psychosocial factors should be encouraged in young type 1 diabetic men with sexual dysfunctions.

## Data Availability

All data generated or analyzed during this study are included in this published article.
